# Azilsartan improves urinary albumin excretion in hypertension mice

**DOI:** 10.18632/aging.205271

**Published:** 2024-03-10

**Authors:** Jun Cao, Dandan Zhang, Wenfeng Li, Wenjin Yuan, Gang Luo, Shaofeng Xie

**Affiliations:** 1 Department of Nephrology, People’s Hospital of Ganzhou, Ganzhou 341001, Jiangxi Province, China

**Keywords:** Azilsartan, urinary albumin excretion, hypertensive nephrosclerosis, KLF2

## Abstract

Hypertension is one of the most important risk factors for chronic kidney diseases, leading to hypertensive nephrosclerosis, including excessive albuminuria. Azilsartan, an angiotensin II type 1 receptor blocker, has been widely used for the treatment of hypertension. However, the effects of Azilsartan on urinary albumin excretion in hypertension haven’t been reported before. In this study, we investigated whether Azilsartan possesses a beneficial property against albuminuria in mice treated with angiotensin II and a high-salt diet (ANG/HS). Compared to the control group, the ANG/HS group had higher blood pressure, oxidative stress, and inflammatory response, all of which were rescued by Azilsartan dose-dependently. Importantly, the ANG/HS-induced increase in urinary albumin excretion and decrease in the expression of occludin were reversed by Azilsartan. Additionally, it was shown that increased fluorescence intensity of FITC-dextran, declined trans-endothelial electrical resistance (TEER) values, and reduction of occludin and krüppel-like factor 2 (KLF2) were observed in ANG/HS-treated human renal glomerular endothelial cells (HrGECs), then prevented by Azilsartan. Moreover, the regulatory effect of Azilsartan on endothelial monolayer permeability in ANG/HS-treated HrGECs was abolished by the knockdown of KLF2, indicating KLF2 is required for the effect of Azilsartan. We concluded that Azilsartan alleviated diabetic nephropathy-induced increase in Uterine artery embolization (UAE) mediated by the KLF2/occludin axis.

## INTRODUCTION

As a common chronic disease worldwide, the incidence of hypertension has been increasing annually in recent years, with a population exceeding 1 billion in the world. In China, the morbidity of hypertension is as high as 12% [1], showing the characteristics of high incidence, high disability rate, high mortality rate, low awareness rate, low cure rate, and low control rate. Hypertension is one of the major risk factors for cardiovascular and cerebrovascular diseases [2]. The kidney is one of the target organs in hypertensive damage and damage to renal structure and function caused by essential hypertension is defined as hypertensive renal disease (HRD), the incidence of which is correlated to the degree and duration of blood pressure elevation [3, 4]. Oxidative stress (OS) is observed in the pathophysiological process of hypertension and is characterized by excessive reactive oxygen species (ROS) production and altered Redox status [5]. Multiple transcription factors are activated by the stimulation of the release of ROS, which further facilitates the production of chemokines and cytokines to induce the recruitment of inflammatory and immune cells, thereby promoting cardiovascular and renal inflammation and fibrosis [6]. The kidney is a hypermetabolic organ, and renal mitochondria are rich in oxidative reactions, making them susceptible to OS injuries. The impaired mitochondrial function is triggered by the produced ROS, which eventually results in the impairment of renal cells and tissue function to aggravate renal damage and the progression of chronic kidney disease [7]. Compared to subjects with normal blood pressure, a long-term increase in blood pressure is observed in hypertensive patients, resulting in continuous increases in intravascular pressure to trigger damage to glomerular capillary endothelial cells, renal tubular epithelial cells, and podocytes, further increasing the basement membrane permeability. The renal filtration barrier system is disrupted and proteins are permeated from blood vessels into the urine, which is characterized by microalbuminuria [8]. Urinary protein is the initiating factor of renal interstitial fibrosis and a risk factor for the development of end-stage renal disease [3, 4]. It is claimed that alleviating OS and inflammatory damage of renal tubular epithelial cells shows a significant repair effect on the renal filtration barrier system [9], which might be a new strategy for HRD treatment.

Kruppel-like factor 2 (KLF2) is a member of the zinc finger family of DNA-binding transcription factors that regulate endothelial cell metabolism. KLF2 expression is found to be increased by shear stress in branched points of blood vessels [10], and it has also been found to inhibit endothelial inflammation [11], which is related to endothelial dysfunction, one of the predictors of atherosclerosis development and cardiovascular events [12]. Also, endothelial dysfunction is a mechanism underlying the development of hypertension [13]. Hypertension is associated with endothelial dysfunction. Bae L, et al. have recently reported the renoprotective effect of KLF2 on glomerular endothelial dysfunction in hypertensive nephropathy through angiotensin II-AT1R [14].

Azilsartan medoxomi is developed by Takeda and belongs to a new generation of selective AT1 subtype angiotensin II receptor antagonist, and is the prodrug of Azilsartan. A clinical study has reported the stable and durable antihypertensive effects of Azilsartan [15]. Apart from its antihypertensive effect, other functions claimed for Azilsartan include the prevention of myocardial hypertrophy [16], vascular remodeling [17], and renal function damage [18]. Furthermore, an extremely suppressive property of Azilsartan against OS and inflammatory damage has been illustrated [19, 20].

In this research, we assumed that Azilsartan may have a protective effect in the increase in uterine artery embolization (UAE) in models of hypertension. The underlying mechanism was further explored.

## MATERIALS AND METHODS

### Animals and treatments

8-week mice were divided into four groups: Control, angiotensin and high-salt (ANG/HS), ANG/HS + Azilsartan (10 μg/day, Cat#:ajci10796, Amgucam, Shanghai, China), and ANG/HS + Azilsartan (20 μg/day) groups for 3 weeks. To construct the ANG/HS model in mice, animals were implanted with an osmotic minipump to administer 60 ng/min ANG for 14 days. During the administration, the HS diet (8% NaCl) was given to mice. After the modeling, mice were dosed with 10 μg/day or 20 μg/day Azilsartan for 3 weeks. The daily water intake, food intake, and mean arterial pressure were detected during the treatments [21].

### Measurement of UAE

Mice were kept in a 24 h metabolic cage for the purpose of collecting urine for 24h while being given free access to water. The collected urine was centrifuged at 3000 rpm for 20 min and the supernatant was retained. The UAE was tested using the ELISA kit (Mlbio, China).

### Enzyme-linked immunosorbent assay (ELISA) for the detection of cytokine levels

50 μL samples were diluted at a 1:1 ratio and loaded into the well, followed by introducing 50 μL Biotin-labeled antibody. After 60 min incubation at 37° C, the reagent was removed and 80 μL HRP-loaded secondary antibody, was followed by half an hour culture at 37° C. Then, 50 μL TMB substrates were added and cultured at 37° C for 10 min, followed by loading 50 μL stop solution and detecting the OD value utilizing a microplate reader (MD-Bio, USA).

### Immunostaining assay

Collected renal tissues were dehydrated by 70%, 80%, and 90% ethanol solution, successively, followed by dehydration with xylene until transparent, which were then embedded and sliced. Slides were incubated with 10% goat serum overnight and were then introduced with the primary antibody against occludin (1:1000, Affinity, USA) at 4° C for 24 hours. The secondary antibody (1:200, Abcam, UK) was then added for incubation at 4° C for 24 hours, and then rinsed and stained with DAB dye. Lastly, images were taken using the inverted microscope (Nikon, Japan).

### Cell culture, treatment, lentiviral KLF2 shRNA transduction

HrGECs were achieved from ATCC (USA) and cultured in Endothelial Cell Medium (ScienCell REsearch Laboratories, USA) supplemented with 10% FBS, 1% endothelial cell growth supplement, and 1% Penicillin/streptomycin (p/s) which were cultured under 37° C and 5% CO_2_. The lentiviral containing a shRNA for KLF2 was transduced into HrGECs for 48 h to obtain the KLF2-silenced HrGECs, which was identified utilizing the Western blotting assay.

### Detection of oxidative stress biomarkers

The content of 4,4’-Methylenedianiline (MDA) and Superoxide dismutase (SOD) activity in renal tissues were checked. A commercial kit (Elabscience, USA) was utilized to examine the MDA content in renal tissues using the TBA method according to the kit instructions. The SOD activity in renal tissues was determined with an EnzyChrom Superoxide Dismutase Assay Kit (BioAssay Systems, USA) according to the kit instructions.

### Fluorescein-5-isothiocyanate (FITC)-dextran assay

HrGECs were loaded on the transwell to form a monolayer, followed by introducing FITC-dextran (AbMole, USA) in the upper chamber. After 60 min culture, the OD value in the lower chamber at 492/520 nm was detected by a microplate reader (MD-Bio, USA).

### Trans-epithelial/endothelial electrical resistance (TEER)

The *in vitro* endothelial permeability was measured with the TEER assay with a 1600R ECIS System (Applied Biophysics, Australia) according to the method described previously [22]. The data was presented with an average of the resistance values (Ω·cm^2^) and the average percent change from baseline TEER.

### Real-time polymerase chain reaction (PCR)

RNAs were extracted from HrGECs using the TRIzol solution, followed by dissolving in ddH_2_O and quantified by detecting the absorption at 260 nm. The transcription to cDNA was performed using the reverse transcription kit (QIAGEN, Germany), followed by conducting the PCR reaction utilizing the ABI 7500 Real-time PCR system (Applied Biosystems, USA) with the SYBR green (Sigma, USA). β-actin is used as the housekeeping gene. The gene levels were calculated with the 2^-ΔΔCt^ method. The primer sequences are listed as below: IL-6: Forward 5’- CACCGGGAACGAAAGAGAAG-3’, Reverse, 5’-TCTGAGGTGCCCATGCTACAT-3’; TNF-α : Forward, 5’-CAGAGGGAAGAGTTCCCCAG-3’, Reverse, 5’-CCTTGGTCTGGTAGGAGACG-3’; IL-1β: Forward, 5’-GCTGCTTCCAAACCTTTGAC-3, Reverse, 5’-AGCTTCTCCACAGCCACAAT-3’; occludin: Forward, 5′-TTGGCTACGGAGGTGGCTATGG-3′, Reverse 5′- ACTAAGGAAGCGATGAAGCAGAAGG-3′; KLF2: Forward, 5’-AGACCTACACCAAGAGTTCGCATC-3′, Reverse 5′-ATC GCACAGATGGCACTGGAATG-3′; β-actin: Forward, 5’-CACCCACTCCTCCACCTTTG-3′, Reverse, 5’-CCACCACCCTGTTGCTGTAG-3′.

### Western blotting assay

Following isolating proteins from HrGECs, the BCA method was performed for the quantification of proteins, which were further separated with a 12% SDS-PAGE. After transferring onto the PVDF membrane, proteins were blocked with skim milk for 2 hours. Subsequently, the membrane was loaded with primary antibodies against occludin (1:1000, Cat#: sc-133256, SCBT, USA), KLF2 (1:1500, Cat#: ab236507, Abcam, USA), or β-actin (1:10000, Cat#: sc-56459, SCBT, USA) for 12 hours at 4° C, followed by adding the secondary antibody (1:2000, Cat#: sc-2357, SCBT, USA) for 1.5 hours [23]. Lastly, the intensity of the blots was quantified using the Bio-Rad Quantity One software (USA) and analyzed using the GraphPad Prism 9 software.

### Statistical analysis

The software GraphPad Prism 9 was used for statistical analysis. Data were presented as mean±standard deviation (S.D.) and the comparison was analyzed using a one-way analysis of variance (ANOVA) method, followed by Bonferroni’s post-hoc test. *P<0.05* was taken as a statistically significant difference.

### Data availability

The data is available at a reasonable request.

## RESULTS

### The effects of Azilsartan on ANG/HS-induced hypertension mice

**Figure 1:**
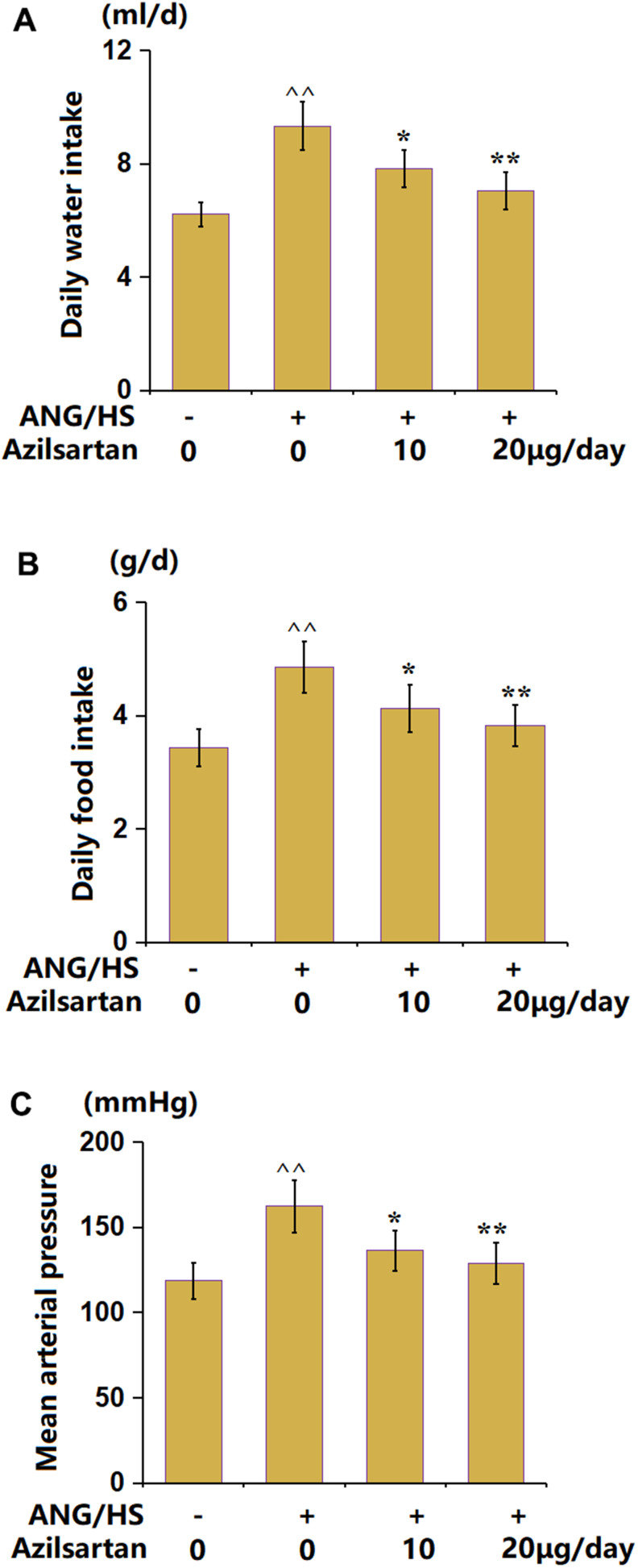
**The effects of Azilsartan on ANG/HS-induced hypertension mice.** (**A**) daily water intake; (**B**) daily food intake; (**C**) Mean arterial pressure (n=6, ^^, P<0.01 vs. vehicle group; *, **, P<0.05, 0.01 vs. ANG/HS group).

After treatments with Azilsartan, the general condition of mice was measured. The daily average water intake (Figure 1A) was increased from 6.22 ml/d to 9.32 ml/d in the ANG/HS group, then reduced to 7.82 ml/d and 7.05 ml/d by 10 and 20 μg/day Azilsartan, respectively. Moreover, the daily food intake values (Figure 1B) in the control, ANG/HS, ANG/HS+Azilsartan (10 μg/day), and ANG/HS+Azilsartan (20 μg/day) groups were 3.43, 4.85, 4.13, and 3.82 g/d, respectively. The mean arterial pressure (Figure 1C) was elevated from 118.5 mmHg to 162.3 mmHg in the ANG/HS group, then reduced to 136.3 mmHg and 128.8 mmHg by 10 and 20 μg/day Azilsartan, respectively. Hypertension symptoms in mice were alleviated by Azilsartan.

### Azilsartan mitigated inflammatory response in renal tissues in ANG/HS- challenged mice

**Figure 2:**
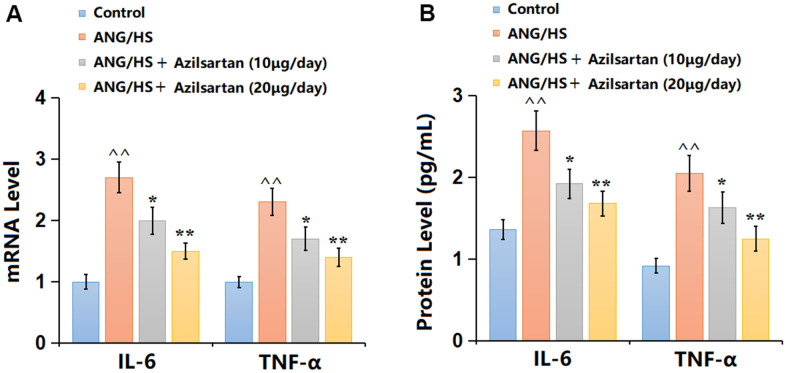
**Azilsartan mitigated inflammatory response in renal tissues in ANG/HS-challenged mice.** (**A**) mRNA levels of IL-6, TNF-α and IL-1β; (**B**) Protein levels of IL-6, TNF-α and IL-1β were measured by ELISA (n=6, ^^, P<0.01 vs. vehicle group; *, **, P<0.05, 0.01 vs. ANG/HS group).

Renal tissues were then extracted for inflammation detection. IL-6, TNF-α, and IL-1β levels were extremely increased in the ANG/HS group, but signally reduced by 10 and 20 μg/day Azilsartan (Figure 2A). Furthermore, the IL-6 level (Figure 2B) in the ANG/HS group was promoted from 1.83 to 3.11 pg/mL, then decreased to 2.38 and 1.78 pg/mL by 10 and 20 μg/day Azilsartan, respectively. Similarly, the TNF-α levels in the control, ANG/HS, ANG/HS+ Azilsartan (10 μg/day), and ANG/HS + Azilsartan (20 μg/day) groups were 1.06, 2.26, 1.76, and 1.37 pg/mL, respectively. Moreover, The expressions of IL-1β in the control, ANG/HS, ANG/HS+ Azilsartan (10 μg/day), and ANG/HS + Azilsartan (20 μg/day) groups were 1.69, 3.58, 2.45, 1.89 pg/mL, respectively. Azilsartan showed a suppressive impact on inflammatory response in ANG/HS-challenged mice.

### Azilsartan attenuated OS in renal tissues in ANG/HS- challenged mice

**Figure 3:**
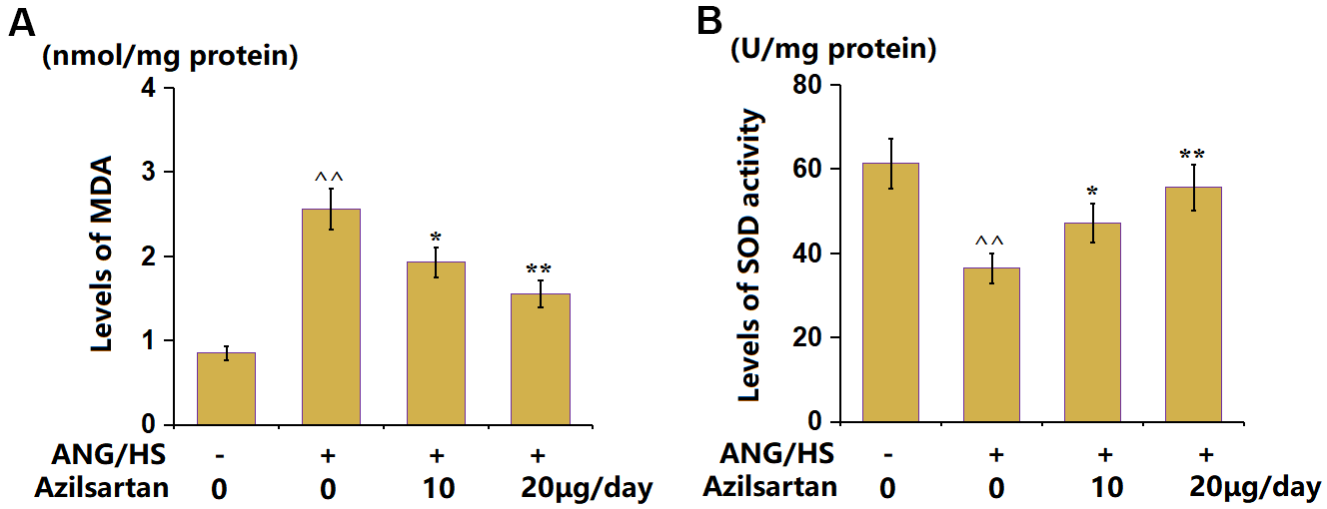
**Azilsartan attenuated oxidative stress in renal tissues in ANG/HS-challenged mice.** (**A**) The levels of MDA in renal tissues; (**B**) The levels of SOD activity in renal tissues (n=5, ^^, P<0.01 vs. vehicle group; *, **, P<0.05, 0.01 vs. ANG/HS group).

The MDA content in ANG/HS- challenged mice was enhanced from 0.85 to 2.56 nmol/mg protein, which was reduced to 1.93 and 1.55 nmol/mg protein by 10 and 20 μg/day Azilsartan, respectively (Figure 3A). In addition, the SOD activity levels (Figure 3B) in the control, ANG/HS, ANG/HS+ Azilsartan (10 μg/day), and ANG/HS+Azilsartan (20 μg/day) groups were 61.3, 36.5, 47.2, and 55.6 U/mg protein, respectively. Azilsartan exerted a suppressive function on OS in ANG/HS-challenged mice.

### The effects of Azilsartan on UAE in ANG/HS-challenged mice

**Figure 4:**
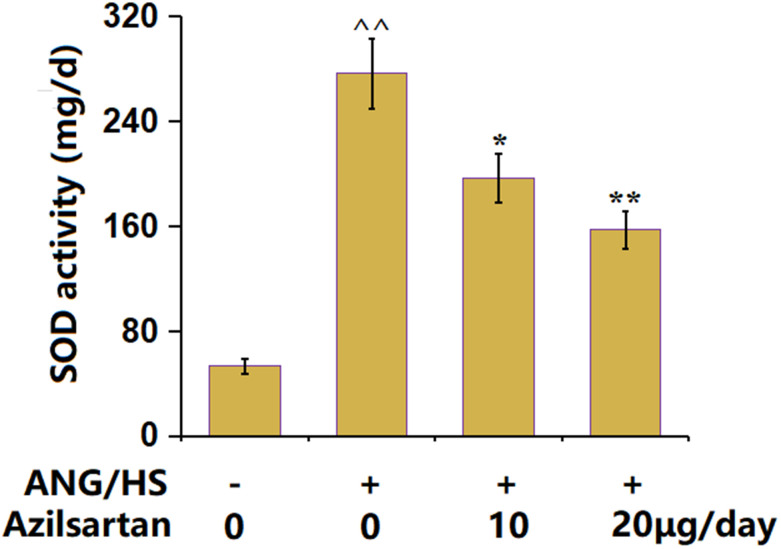
The effects of Azilsartan on urinary albumin excretion in ANG/HS-challenged mice (n=6, ^^, P<0.01 vs. vehicle group; *, **, P<0.05, 0.01 vs. ANG/HS group).

The UAE in ANG/HS-challenged mice was found to be markedly increased from 53.5 mg/d to 276.3 mg/d, which signally declined to 196.5 mg/d and 157.2 mg/d by 10 μg/day and 20 μg/day Azilsartan (Figure 4), implying a repairment by Azilsartan on UAE in ANG/HS-challenged mice.

### The effects of Azilsartan on the expression of occludin in renal tissues in ANG/HS-challenged mice

**Figure 5:**
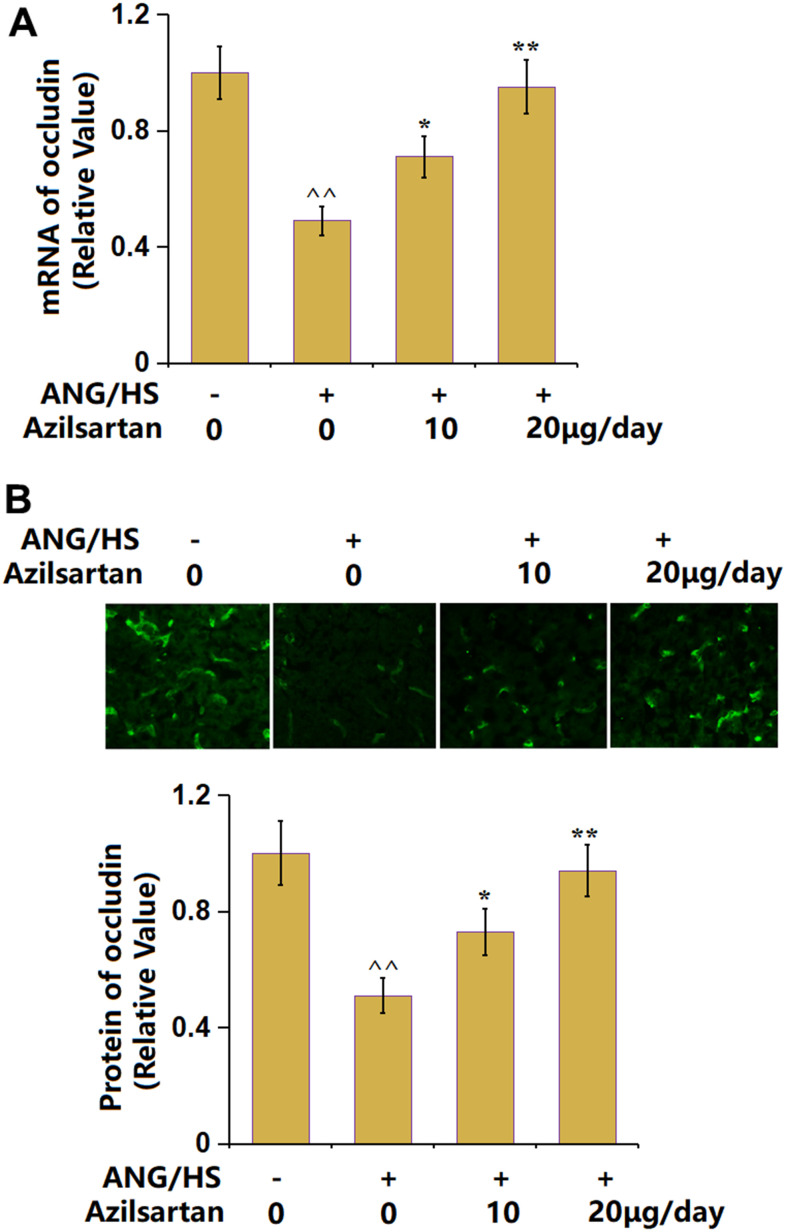
**The effects of Azilsartan on the expression of occludin in renal tissues in ANG/HS-challenged mice.** (**A**) mRNA of occludin; (**B**) Protein of occludin as measured by immunostaining. Scale bar, 100 μm (n=5 or 6, ^^, P<0.01 vs. vehicle group; *, **, P<0.05, 0.01 vs. ANG/HS group).

Occludin is a critical tight junction (TJ) protein in renal tubular epithelial tissues [24]. Occludin was extremely downregulated in renal tissues of ANG/HS- challenged mic**e**, which was signally rescued by 10 μg/day and 20 μg/day Azilsartan (Figure 5A, 5B), suggesting an elevation property of Azilsartan on the occludin level in renal tissues of ANG/HS-challenged mice.

### Azilsartan ameliorated ANG/HS-induced increases in endothelial monolayer permeability in HrGECs

**Figure 6:**
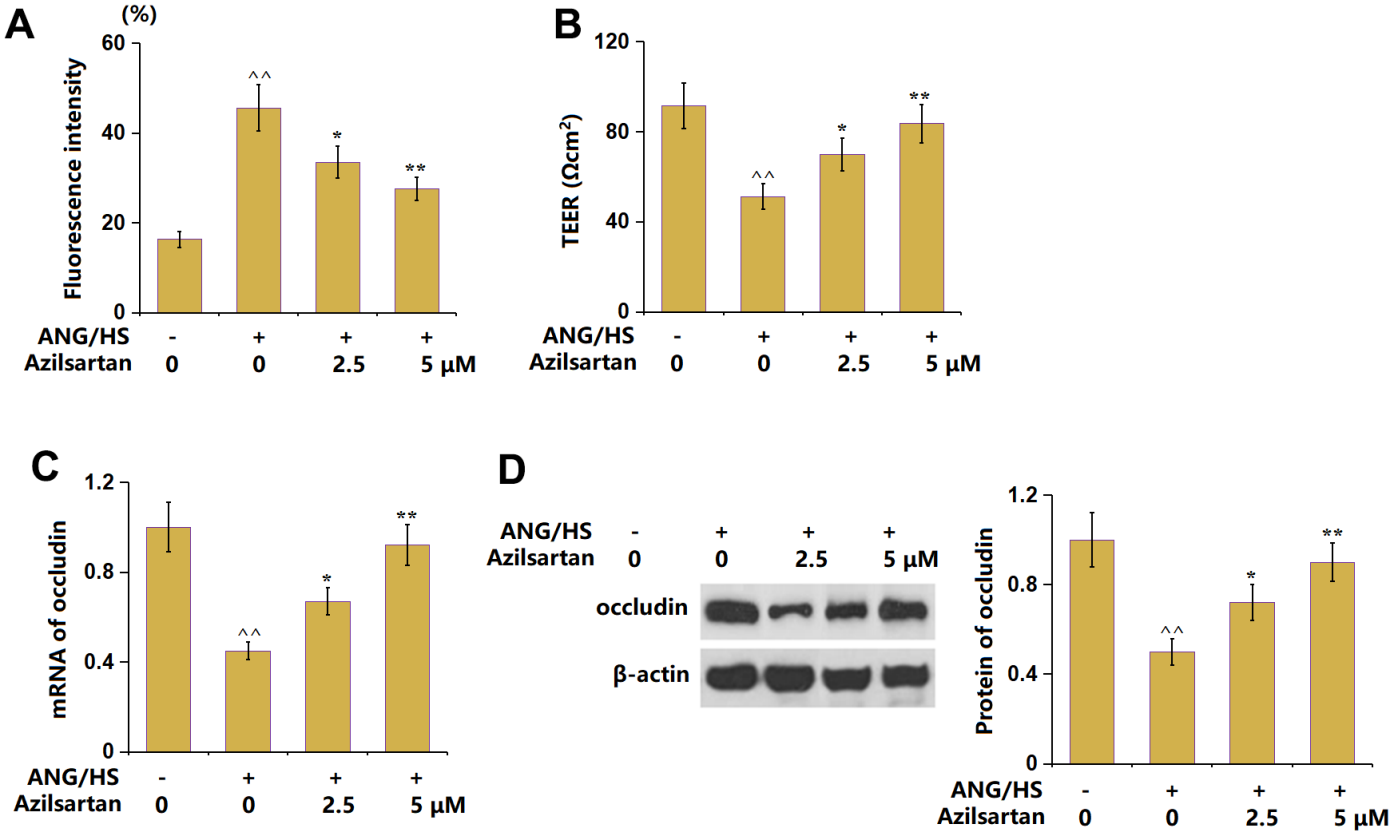
**Azilsartan ameliorates ANG/HS-induced increase in endothelial monolayer permeability in human renal glomerular endothelial cells (HrGECs).** HrGEC monolayer was treated with ANG/HS with or without Azilsartan (2.5, 5 μM) for 24 hours. (**A**) Fluorescence intensity of FITC-dextran; (**B**) Trans-endothelial electrical resistance (TEER) was assayed; (**C**) mRNA of occludin; (**D**) Protein levels of occludin (n=5, ^^, P<0.01 vs. vehicle group; *, **, P<0.05, 0.01 vs. ANG/HS group).

To probe the functional mechanism, an HrGECs monolayer was constructed and treated with ANG/HS with or without Azilsartan (2.5, 5 μM) for 24 hours. The fluorescence intensity of FITC-dextran in the ANG/HS-challenged HrGECs monolayer (Figure 6A) was increased from 16.3% to 45.6%, which was signally decreased to 33.5% and 27.6% by 2.5 and 5 μM Azilsartan, respectively. Furthermore, the TEER values (Figure 6B) in the control, ANG/HS, 2.5 μM Azilsartan, and 5 μM Azilsartan groups were 91.5, 51.2, 69.8, and 83.5 Ωcm^2^, respectively. Moreover, the occludin level (Figure 6C, 6D) in ANG/HS- challenged HrGECs was markedly repressed, but observably rescued by 2.5 and 5 μM Azilsartan. The increased endothelial monolayer permeability in HrGECs was ameliorated by Azilsartan.

### Azilsartan prevents ANG/HS-induced reduction in KLF2 in HrGECs

**Figure 7:**
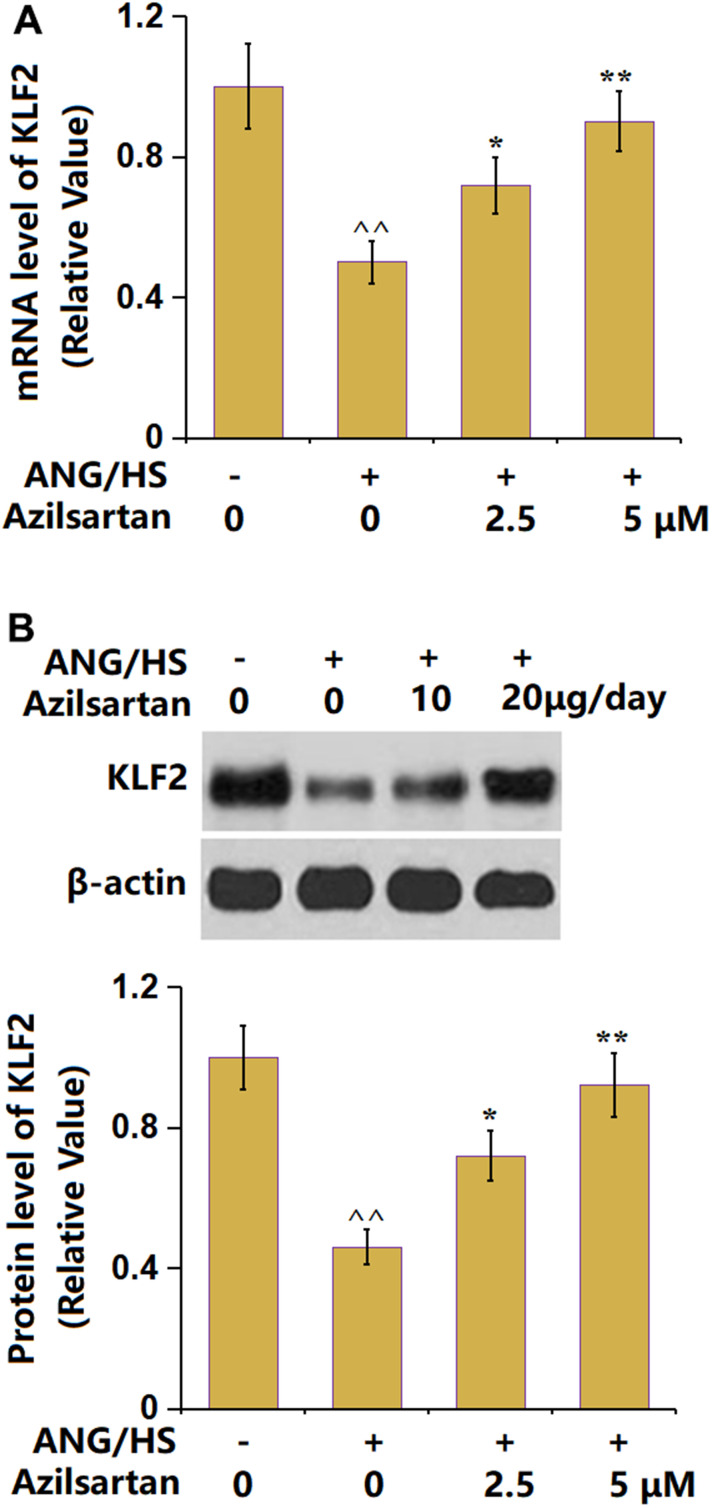
**Azilsartan prevents ANG/HS-induced reduction in KLF2 in human renal glomerular endothelial cells (HrGECs).** HrGEC monolayer was treated with ANG/HS with or without Azilsartan (2.5, 5 μM) for 24 hours. (**A**) mRNA level of KLF2; (**B**) Protein level of KLF2 (n=6, ^^, P<0.01 vs. vehicle group; *, **, P<0.05, 0.01 vs. ANG/HS group).

KLF2 is a regulatory transcriptional factor mediating TJ protein levels [25]. KLF2 (Figure 7) was found memorably downregulated in the ANG/HS-challenged HrGECs monolayer but signally upregulated by 2.5 and 5 μM Azilsartan, implying an involvement of KLF2 in the function of Azilsartan.

### Silencing of KLF2 abolished the protective effects of Azilsartan against ANG/HS-induced aggravation of endothelial permeability in HrGECs

**Figure 8:**
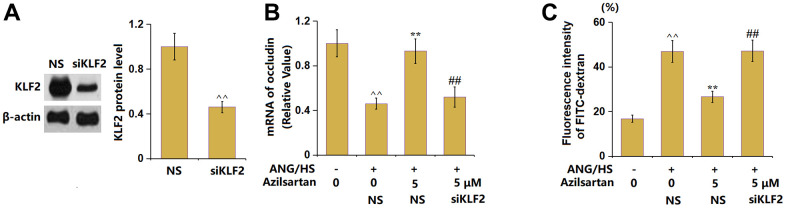
**Silencing of KLF2 abolished the protective effects of Azilsartan against ANG/HS-induced aggravation of endothelial permeability in HrGECs.** HrGEC monolayer was transduced with lentiviral KLF2 shRNA, followed by stimulation with ANG/HS with or without Azilsartan (5 μM) for 24 hours. (**A**) Western blot revealed successful knockdown of KLF2; (**B**) mRNA level of occludin; (**C**) Fluorescence intensity of FITC-dextran (n=6, ^^, P<0.01 vs. vehicle group; **, P<0.01 vs. ANG/HS group; ##, P<0.01 vs. ANG/HS+ Azilsartan group).

To provide evidence for the involvement of KLF2 in the function of Azilsartan, the HrGECs monolayer was transduced with lentiviral KLF2 shRNA, followed by stimulation with ANG/HS with or without Azilsartan (5 μM) for 24 hours. The silencing of KLF2 in HrGECs was identified by the results shown in Figure 8A. The declined occludin level observed in ANG/HS- challenged HrGECs was markedly elevated by Azilsartan, which was signally reversed by knocking down KLF2 (Figure 8B). Furthermore, the fluorescence intensity of FITC-dextran in the ANG/HS-challenged HrGECs monolayer (Figure 8C) was promoted from 16.8% to 46.9%, which was notably reduced to 26.6% by Azilsartan. After the knockdown of KLF2, the fluorescence intensity was reversed to 47.2%.

## DISCUSSION

Previous studies on HRD mainly focused on the glomerulus. However, recent researches have claimed that the degree of renal tubulointerstitial damage is more associated with renal function [26]. Some scholars have shown that reducing the functional damage of renal tubular epithelial cells is beneficial for the repair of renal damage, thereby reducing or partially inhibiting renal interstitial fibrosis [27]. OS affects hypertension rats by aggravating the severity of renal damage, suggesting that OS shows an intrinsic and inevitable relationship with hypertension [28]. Renal tubular dysfunction is reported to be relevant to OS activation. A sharp decline of SOD activity is triggered by accumulated ROS, while MDA is increased substantially and NO is inactivated, resulting in the reduced bioavailability of NO. After the oxidation of arachidonic acid in the kidney, a large number of prostaglandins are synthesized, which has a direct vasoconstriction effect to further aggravate renal damages [29]. Similar to data reported by Bakri [30], extremely increased water intake, food intake, and arterial pressure were observed in ANG/HS-challenged mice, all of which were signally rescued by Azilsartan, suggesting an alleviative effect of Azilsartan on ANG/HS-induced hypertension in mice. Furthermore, activated OS and enhanced cytokine production were observed in ANG/HS- challenged mice, which were also claimed in cardiac tissues of ANG/HS-challenged mice by Yang [31]. The curative function of Azilsartan on ANG/HS- challenged mice was accompanied by the alleviation of OS and inflammation, implying a correction between the function of Azilsartan and OS inhibition.

Epithelial junction complexes include TJs, adhere junctions (AJs), and gap junctions. TJs are located at the top of all junction complexes, with AJs located underneath for the apical junction complex [32]. TJs are characterized as the banded structure circled epithelial cells, which divide membranes into the apical and basolateral parts. The polarity, proliferation, and differentiation of epithelial cells can be regulated by proteins in TJs [33]. Occludin is the first transmembrane protein discovered in TJs, which participates in maintaining the permeability of TJs and the integrity of the epithelial cell barrier [34]. In ANG/HS- challenged mice, consistent with Bakri’s report [30], an increased UAE was observed, suggesting disruption of the integrity of the epithelial cell barrier. Furthermore, the occludin level was found to be reduced, which explained the increased TJs permeability. The curative function of Azilsartan on ANG/HS-challenged mice was accompanied by reduced UAE and increased occludin levels, implying that the function of Azilsartan was correlated with the integrity of epithelial cell barrier. Our *in vitro* study revealed that the permeability of the HrGECs monolayer was increased by ANG/HS, also observed in high glucose-stimulated HrGECs [35]. The reparative effect of Azilsartan on the permeability of the HrGECs monolayer was accompanied by the increased occludin level, implying that the function of Azilsartan was correlated with the upregulation of occludin.

KLF2 was discovered by Anderson in 1995 and is mainly expressed in lung tissues [36]. As a member of the KLF family, KLF2 has the common structural features of the family, including a DNA zinc finger binding domain, transcription activation domain, and autoinhibitory domain, among which the autoinhibitory domain is adjacent to the zinc finger domain [37]. Studies have found that KLF2 has specific functions in the maintenance of normal vascular function, adipocyte differentiation, and stemness maintenance of embryonic stem cells [38, 39]. Recently, KLF2 has been claimed to regulate the level of TJ proteins, including occludin [40]. In our research, the KLF2 level was found to be extremely reduced in ANG/HS- challenged HrGECs, then rescued by Azilsartan, implying that the function of Azilsartan was correlated with the upregulation of KLF2. Furthermore, the influence of Azilsartan on the occludin level and endothelial permeability were abrogated by the silencing of KLF2, which further identified the mediation of KLF2 in the function of Azilsartan. In future studies, the direct target of Azilsartan in HrGECs will be probed to fully understand the functional mechanism of Azilsartan in treating HRD.

Collectively, Azilsartan alleviated ANG/HS-induced increase in UAE mediated by the KLF2/occludin axis.
